# Non-oral continuous drug delivery based therapies and sleep dysfunction in Parkinson’s disease

**DOI:** 10.1007/s00702-023-02640-7

**Published:** 2023-05-01

**Authors:** P. Tall, M. A. Qamar, L. Batzu, V. Leta, C. Falup-Pecurariu, K. Ray Chaudhuri

**Affiliations:** 1 Department of Basic and Clinical Neurosciences, Institute of Psychiatry, Psychology and Neuroscience, King’s College London, 16 De Crespigny Park, London, SE5 8AB UK; 2 Parkinson’s Foundation Centre of Excellence, King’s College Hospital, London, UK; 3 Faculty of Medicine, Transilvania University, Brasov, Romania; 4 Department of Neurology, County Clinic Hospital, Brasov, Romania

**Keywords:** Continous drug delivery, Parkinsons, Levodopa, Apomorphine, LCIG, LECIGON, Sleep, Park-sleep

## Abstract

Continuous drug delivery (CDD) has emerged as a feasible and pragmatic therapeutic option for dopamine replacement therapy in advanced Parkinson’s disease (PD). CDD aims to mimic the physiological tonic dopamine release from striatal dopaminergic neurons and thus reduces the severity and duration of motor and non-motor fluctuations partly related to pulsatile levodopa stimulation. Non-motor symptoms and fluctuations are ubiquitous in PD and include sleep dysfunction, a problem that occurs in over 90% of PD patients across all stages, from prodromal to palliative. In this review, we discuss the currently available and in development non-oral dopaminergic CDD strategies with a focus on their efficacy in the treatment of the burdensome sleep dysfunction in PD.

## Introduction

Parkinson’s disease (PD) is the second fastest growing neurodegenerative disorder in the world, projected to double in numbers worldwide by 2060 and now recognised as a progressive multisystem syndrome consisting of both motor and non-motor features (Marras et al. 2018; Chaudhuri et al. 2013). In advanced PD, the syndromic nature of the condition emerges, and conventional oral management options become challenging and non-optimally effective, in part due to barriers to oral absorption of drugs (Leta et al. 2023) but also because of their associated dopaminergic pulsatile stimulation (Leta et al. 2019). This is where non-oral continuous drug delivery (CDD) methodology has shown to provide an encouraging and successful management strategy, with a wealth of data suggesting consistent motor as well as non-motor benefits (Chaudhuri et al. 2013; Leta et al. 2023; Leta et al. 2019). The data are particularly strong in relation to intrajejunal levodopa infusion (IJLI), as is evident from the Global Long-term Registry on Efficacy and Safety of LCIG (GLORIA) and Duoglobe registry datasets (Antonini et al. 2017).

### Continuous drug delivery in Parkinson’s

Levodopa is the gold-standard treatment for motor features of PD but, with the progressive nature of PD, the effectiveness of levodopa diminishes over 5–10 years with development of motor fluctuations and dyskinesia (Connolly and Lang 2014).

Gastrointestinal (GI) dysfunction in PD can occur in all stages of the disease (Leta et al. 2021a; Leta et al. 2021b; Leta et al. 2023; Metta et al. 2022; Ray Chaudhuri, et al. 2016). It has been suggested that GI dysfunction is in part responsible for motor fluctuations such as delayed or no-ON, erratic absorption and consequent dyskinesias (Leta et al. 2023). There are several other pathophysiological mechanisms behind the development of both motor and non-motor fluctuations including the progressive nature of PD and the pulsatile delivery of common oral dopaminergic strategies, however, it is beyond the scope of this review to discuss these in detail (Leta et al. 2019). Focus on non-oral delivery of levodopa, to bypass the dysfunctional GI tract, is proposed and proven to be the next step in adequate control of PD symptomology (Güneş and Karavana 2022).

There are several different classes of CDD therapies currently available, as is shown in Table 1, including infusion therapies such as the Levodopa-Carbidopa intestinal gel (LCIG), Levodopa-carbidopa-entacapone intestinal gel, Apomorphine subcutaneous infusion, and the Rotigotine (RTG) transdermal patch. New strategies, such as the subcutaneous infusion of Levodopa, aiming to optimise the delivery and half-life of the pharmacological intervention, are currently under development (Ray Chaudhuri et al. 2016; Wamelen et al. 2018). This review will focus primarily on these non-oral CDD therapies given their increasing use in clinical practise globally and their effect on sleep dysfunction.

**Table 1:** Summary of non-oral continuous drug delivery systems discussed

Name	Formula	Development phase	CDD method	Evidence for sleep improvement
Non-oral levodopa-based CDD				
LCIG	Levodopa Carbidopa	2005 EMA approved; 2015 FDA approved	PEG/J	Strong
LECIG	Levodopa Entacapone Carbidopa	2018 Sweden approved; 2021 other Europe	PEG/J	Minimal
ND0612	Levodopa Carbidopa	Currently phase III (NCT04006210)	SC	Minimal
ABBV-951	Foslevodopa Foscarbidopa	Currently phase III (NCT04380142)	SC	Pending
IPO-001	Levodopa Carbidopa	Currently phase I (NCT03419806)	SC/ IV	Pending
Non-oral non-Levodopa-based CDD				
RTG patch	Non-ergot dopamine agonist	2006 EMA approval; 2007 FDA approval	Transdermal	Moderate-strong
APO-pump	Non-ergot dopamine D2 agonist	NDA approved, ongoing trials	PEG/J	Moderate-strong
ND0701	Non-ergot dopamine D2 agonist	Currently phase I/II	Transdermal	Pending
SER-214	Non-ergot dopamine agonist	Currently phase I (NCT02579473)	Transdermal	Pending

We classify evidence for sleep improvement based on number of evidence to that effect, adaptation of the previously suggested level of evidence ranking (Hussein et al. 2018; Teunissen et al. 2015). *Minimal* is classed as less than 2 articles supporting notion of sleep improvement, with more than 4 conflicting studies. *Moderate* is classed as 3–4 articles supporting the notion of sleep improvement, with less than 4 conflicting studies. *Mod-Strong* is classed as 5–6 articles supporting the notion of sleep improvement, with less than 3 conflicting studies. *Strong* is classed as 7 or more studies supporting the notion of sleep improvement, with less than 2 conflicting studies. *Pending* is classed as no evidence currently available

*APO* apomorphine, *CDD* continuous drug delivery, *EMA* European medicine agency, *FDA* Food and drug administration, *IV* intravenous, *LCIG* levodopa-carbidopa intestinal gel, *LECIG* levodopa-entacapone-carbidopa intestinal gel, *PEG/J* percutaneous endoscopic transgastric jejunostomy, *RTG* rotigotine, *SC* subcutaneous

### Continuous drug delivery and sleep dysfunction

NMS burden and non-motor fluctuations have shown to increase with PD progression with advanced stages manifesting a difficult conundrum for many clinicians (Brun et al. 2014). There is a wide range of evidence (Chaudhuri et al. 2013; Chaudhuri et al. 2022a; Antonini et al. 2017; Wamelen et al. 2018) demonstrating that a variety of CDD strategies ameliorate NMS burden, with a consistent beneficial effect on sleep as reported in open label and randomised studies (Chaudhuri et al. 2013, 2022a; Antonini et al. 2017; Rosa-Grilo et al. 2017).

Sleep dysfunction is reported by 67–98% of people with Parkinson’s disease (PwP) (Medcalf 2005). Sleep dysfunction describes a spectrum of different sleep problems including disturbances of sleep, such as insomnia and rapid eye movement (REM) behaviour disorder (RBD), and disturbances of wakefulness such as excessive daytime sleepiness (EDS) (Falup-Pecurariu and Diaconu 2017). NMS burden progresses with the disease, amid sleep disturbances being, not only a common NMS in advance stages of PD but also shown to be an independent predictor of NMS burden itself (Kurlawala et al. 2021; Santos-García, et al. 2021; Dhawan et al. 2006). CDD may be beneficial in the tackling of sleep dysfunction in PD, due to the continuous delivery of dopamine throughout the night, by not only improving nocturnal motor complications, but also by improving sleep structure and reducing sleep fragmentation (Raeder et al. 2021).

## Currently available levodopa-based strategies

CDD, in the form of levodopa intestinal infusion, may provide a stable plasma level of levodopa, thereby relieving both motor and non-motor fluctuations face with oral delivery alone (Antonini et al. 2017; Chaudhuri et al. 2022a; Thakkar et al. 2021). Levodopa intestinal infusion is available in current clinical practice globally, delivered as a pump-based system.

***Levodopa-carbidopa intestinal gel (LCIG)***: a stable gel suspension, is delivered continuously via a percutaneous gastrojejunostomy and portable pump hence delivering levodopa directly to the jejunum, bypassing the GI-related absorption issues (Leta et al. 2023; Ray Chaudhuri et al. 2016; Chaudhuri et al. 2022a). LCIG has already been shown to be effective in reducing dyskinesias and other motor features in advanced PD (Standaert et al. 2017), but LCIG has robustly demonstrated to be a safe and effective option in improving sleep quality as a burdensome NMS (Honig et al. 2009). A meta-analysis study, with over 1200 advanced staged PwP, report LCIG having significantly improved NMS burden, particularly sleep outcomes, such as EDS, insomnia and nocturia, leading the authors to conclude that LCIG is an effective treatment option for advanced PwP, which can concurrently be an effective treatment for sleep burden in these cases (Chaudhuri et al. 2022a). Whilst we do not yet fully understand the pathophysiology for these improvements, Positron Emission Tomography (PET) studies have pointed to a reduction in dopaminergic D2 receptors in the hypothalamus which may provide the link between sleep disturbances in PD and dopaminergic dysfunction (Politis et al. 2008). Furthermore, LCIG was observed to have reduced both night-time and day-time sleep issues such as insomnia and EDS, respectively, as measured by PD Sleep Scale 2 (PDSS-2) and Epworth Sleepiness Scale (ESS) (Trenkwalder et al. 2011a; Kendzerska et al. 2014). Several other studies have corroborated these findings, including the GLORIA study, which found that LCIG led to persistent noteworthy reductions in NMS burden throughout its 24-month duration, with sleep/fatigue disturbances demonstrating the greatest improvement (Antonini et al. 2017). Furthermore, the currently ongoing DUOGLOBE study found significant improvements in multiple different NMS domains, including quality of sleep and daytime somnolence at a 12-month follow-up, while the results of the planned 24 and 36-month follow-up assessments may provide further understanding of this LCIG-driven changes in NMS burden over time (Standaert et al. 2021). In summary, LCIG has proven to not only be an effective therapy option for advanced PwP for motor control but there is ample evidence to show its benefit on NMS burden, specifically sleep issues.

***Levodopa-carbidopa-entacapone intestinal gel (LECIG) infusion***: it has been recently developed, increasing the bioavailability of levodopa further (Nyholm and Jost 2022; Senek et al. 2017, 2020; Öthman, et al. 2021). In 2018, Sweden was first to gain approval to use LECIG, which has subsequently now been approved for market in other European countries (Nyholm and Jost 2022; Senek et al. 2017). No formal study exploring NMS, let alone sleep, with LECIG application has been documented, although an ongoing international longitudinal registry of LECIG users, ELEGANCE, will ideally provide some initial information on effect of LECIG on NMS and by default, sleep (Nyholm and Jost 2022).

***LCIG used in combination with opicapone***: a long acting, third generation COMT inhibitor, was also demonstrated to reduce costs of this device-aided therapy (Leta et al. 2020). Open label studies have already suggested that both entacapone and tolcapone might improve sleep dysfunction and NMS burden in PwP (Muhlack et al. 2014; Park et al. 2020) and a recent retrospective open label study suggest that introduction of opicapone in real-life PwP with motor fluctuations stabilises NMS burden and aspects of sleep dysfunction after one year, while use of entacapone was associated with a worsening of the above-mentioned aspects. There are no formal studies on the use of LCIG with opicapone to address NMS or sleep issues. However, a small open label study is currently investigating the effect of oral levodopa and opicapone on sleep dysfunction (the OpiSleep study) and, if positive, it may support the use of LCIG with opicapone for night-time benefit in PD, particularly where it is not possible to use IJLI for 24 h because of various difficulties, including cost issues (Leta et al. 2020; Santos García et al. 2022; Chaudhuri et al. 2022b).

## Levodopa-based strategies in development

***ABBV-951***: a new formulation of levodopa and carbidopa prodrugs (foslevodopa/foscarbidopa), is delivered through a continuous subcutaneous infusion delivery system connected to a pump (Rosebraugh et al. 2021; Soileau et al. 2022; Aldred et al. 2022). Phase I trials have shown promising results of lower motor fluctuations, with further promising results from recent phase III trials finding improvement in NMS, especially sleep disturbances (Soileau et al. 2022). ABBV-951 permits the delivery of varying levodopa doses, in smaller volumes than is seen in LCIG and, unlike LCIG, surgery is not required to implant the delivery device, making it an ideal non-surgically invasive CDD therapy option (Soileau et al. 2022). Ongoing longitudinal studies will work further to provide more understanding on ABBV-951 impact on NMS and sleep.

***ND0612***: a formulation of levodopa-carbidopa, is another continuous infusion administered via a pump which is fed through a cannula inserted into the subcutaneous layer of the skin (Giladi et al. 2021; LeWitt 2022). Although there is positive motor data from phase I and II trials, there has been little data published on its effect on NMS, specifically sleep dysfunction (Giladi et al. 2021). Despite this, the limited available data on ND0612-treated PwP have indicated improvement in sleep quality during phase I and II trials, as assessed through PDSS (Giladi et al. 2021).

***IPO-001 (Infudopa***): levodopa-carbidopa delivered through subcutaneous (DIX102) or intravenous (DIX101) route, is currently under phase I trials (NCT03419806) (Ray Chaudhuri et al. 2016; Larson and Simuni 2022; Bergquist et al. 2022) and there is no data on sleep or NMS.

## Currently available non-levodopa based strategies

***Rotigotine (RTG) transdermal patch***: the first widely available transdermal therapy for PwP, is a treatment option with potential in all stages of PD (Raeder et al. 2021). RTG is a non-ergot dopamine agonist, applied using a silicon-based patch, activating dopaminergic (D1-5) receptors, alongside having high affinity for selected serotonergic (5HT_1A_) and adrenergic (α_2_) receptors (Wood et al. 2015; Jenner and Katzenschlager 2016; Chen et al. 2009). RTG patch has shown to provide stable extracellular levels, achieving continuous dopaminergic stimulation, and subsequently reducing motor fluctuations and dyskinesia (Raeder et al. 2021; Kehr et al. 2007; Zhou et al. 2013). Furthermore, RTG patch’s efficacious impact on sleep disturbances in PwP has been echoed by several studies as confirmed using sleep-specific scales (Rosa-Grilo et al. 2017; Giladi et al. 2010; Fei et al. 2019; Bhidayasiri et al. 2017; Pagonabarraga et al. 2015; Ceballos-Baumann and Häck 2011; Pierantozzi et al. 2016). The RECOVER study highlighted improvements in nocturnal sleep disturbances, such as nocturnal akinesia, RLS, immobility, cramps, tremor and nocturnal pain, alongside dopaminergic daytime NMS (Trenkwalder et al. 2011b). In placebo-controlled trials RTG patch has not replicated the significant improvement witnessed previously, however in such trials the participants were early-PD with advance PD not included, which does not reflect the burden RTG patch has shown to overcome in advance PD (Antonini et al. 2015). Finally, overnight RTG transdermal patch in combination with LCIG infusion is well tolerated in advanced PD and can further improve NMS (Lau et al. 2022).

***Other application of RTG***: including the combination of RTG with biodegradable bioconjugate polymers or polyoxazolines, may enhance the control of drug delivery and increase the half-life of RTG (Ray Chaudhuri et al. 2016). One such example includes SER-214 (NCT02579473), which has entered a phase 1 study in de novo PwP (Güneş and Karavana 2022).

***Apomorphine subcutaneous infusion***: a non-ergoline dopamine D2 agonist, is known to provide a positive impact on motor features, but also NMS of PD (Trenkwalder et al. 2015; García Ruiz et al. 2008; Martinez-Martin et al. 2011; Martinez-Martin et al. 2015; Borgemeester et al. 2016; Chaudhuri and Schapira 2009). Administration of nocturnal Apomorphine infusion has led to significant reduction in nocturnal complications (Chaudhuri and Leta 2022; Cock et al. 2022; Reuter et al. 1999). Of note, the APORMORPHEE study found a significant change in mean PDSS score, in comparison to controls, leading the authors to conclude subcutaneous night-time infusion of Apomorphine may be useful in the treatment of insomnia and other sleep disturbances in advanced PwP (Cock et al. 2022). The efficacy of nocturnal Apomorphine infusion in ameliorating RLS symptoms was also investigated, demonstrating a reduction in nocturnal discomfort, leg movement and spasms (Reuter et al. 1999). Delays in time-to-ON with levodopa are another frequent complication, known as morning akinesia, for which apomorphine has also been indicated to have a rapid improvement in time-to-ON in PwP who experience motor and non-motor fluctuations (Trenkwalder et al. 2015; Isaacson et al. 2017; Stacy and Silver 2008). The results of studies such as these suggest apomorphine can successfully overcome refractory nocturnal complications in both selected RLS patients and PwP.

## Non-levodopa based strategies in development

***Other applications of RTG***: including the combination of RTG with biodegradable bioconjugate polymers or polyoxazolines, may enhance the control of drug delivery and increase the half-life of RTG (Ray Chaudhuri et al. 2016). One such example includes SER-214 (NCT02579473), which has entered a phase 1 study in de novo PwP (Güneş and Karavana 2022).

***Apomorphine pump patch***: the ND0701, is currently in Phase 1 trials (Ramot et al. 2018). Results thus far yield ND0701 as a safe and well-tolerated CDD (Ramot et al. 2018; Carbone et al. 2019), with its bioavailability matching that of standard apomorphine infusion, however, we do not currently have any data indicating ND0701s efficacy in the treatment of motor or NMS, and as a result, are not aware of its impact on sleep dysfunction.

## Conclusion

**Fig. 1:**
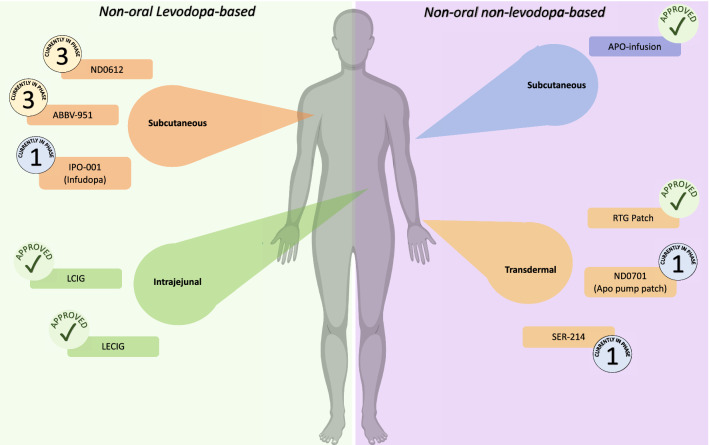
Overview of non-oral continuous drug delivery strategies, currently available and under development, in Parkinson’s disease. *LCIG* levodopa-carbidopa intestinal gel, *LCECI*G levodopa-carbidopa-entacapone intestinal gel, *RTG* rotigotine, *APO* apomorphine

CDD therapies are fundamental in providing symptomatic relief of both motor and NMS in advanced stages of PD, and in the instance of refractory symptoms. Therapies such as those outlined in this review are crucial in overcoming complications related to disease progression and the use of standard oral interventions, including GI dysfunction, motor fluctuations and dyskinesia. The developments to date in both levodopa-and non-levodopa based continuous strategies represent an exciting achievement in the effective management of PD symptoms (Fig. 1), particularly in the case of the burdensome sleep dysfunction. Alongside the successful amelioration of PD symptoms, the use of CDD provides many options for clinicians, including simplification of medication regime.

This review outlines the current knowledge on CDD and sleep and, in particular, of its ability to provide some relief to PwP who have advanced PD with sleep dysfunction. However, further research is needed to evaluate these CDD modalities against each other and other oral continuous therapies.

## Data Availability

The authors confirm that the data supporting the findings of this study are available within the article with references.
